# Bridging the Gap Between Training and Competition in Elite Rink Hockey: A Pilot Study

**DOI:** 10.1177/19417381241273219

**Published:** 2024-08-27

**Authors:** António Ferraz, Enrique Alonso Pérez-Chao, João Ribeiro, Konstantinos Spyrou, Tomás T. Freitas, João Valente-dos-Santos, Pedro Duarte-Mendes, Pedro E. Alcaraz, Bruno Travassos

**Affiliations:** Center in Sports Science, Health Sciences and Human Development (CIDESD), Department of Sport Sciences, University of Beira Interior, Covilhã, Portugal, and CIFD, Sports Research, and Training Center, Jean Piaget University of Angola, Luanda, Angola; Faculty of Sports Sciences, University Alfonso X el Sabio, Villanueva de la Cañada, Spain; Center in Sports Science, Health Sciences and Human Development (CIDESD), Department of Sport Sciences, University of Beira Interior, Covilhã, Portugal, and Polytechnic Institute of Guarda, School of Education, Communication and Sports, Guarda, Portugal; UCAM Research Center for High Performance Sport, UCAM Universidad Católica de Murcia, Murcia, Spain, Facultad de Deporte, UCAM Universidad Católica de Murcia, Murcia, Spain, and SCS, Strength and Conditioning Society, Murcia, Spain; UCAM Research Center for High Performance Sport, UCAM Universidad Católica de Murcia, Murcia, Spain, Facultad de Deporte, UCAM Universidad Católica de Murcia, Murcia, Spain, SCS, Strength and Conditioning Society, Murcia, Spain, and NAR - Nucleus of High Performance in Sport, São Paulo, Brazil; CIDEFES, Centre for Research in Sport, Physical Education, Exercise and Health, Lusófona University, Lisboa, Portugal and COD, Center of Sports Optimization, Sporting Clube de Portugal, Lisbon, Portugal; Department of Sport and Well Being, Polytechnic Institute of Castelo Branco, Castelo Branco, Portugal, and Sport, Health and Exercise Research Unit - SHERU, Polytechnic Institute of Castelo Branco, Castelo Branco, Portugal; UCAM Research Center for High Performance Sport, UCAM Universidad Católica de Murcia, Murcia, Spain, Facultad de Deporte, UCAM Universidad Católica de Murcia, Murcia, Spain, and SCS, Strength and Conditioning Society, Murcia, Spain; Center in Sports Science, Health Sciences and Human Development (CIDESD), Department of Sport Sciences, University of Beira Interior, Covilhã, Portugal, and Portugal Football School, Portuguese Football Federation, Lisbon, Portugal

**Keywords:** elite athletes, load monitoring, performance analysis, team sports, tracking systems

## Abstract

**Background:**

Monitoring training load and competition load is crucial for evaluating and improving athlete performance. This study proposes an applied approach to characterize and classify the training task specificity in relation to competition in a top-level rink hockey team, considering external and internal load from training tasks and competition.

**Hypothesis:**

Training tasks and game demands have significant dose-response differences, and exercises can be classified successfully based on their physiological and biomechanical demands.

**Study Design:**

Cross-sectional study.

**Level of Evidence:**

Level 5.

**Methods:**

Ten elite-level male rink hockey players participated in this study. Players were monitored on 6 different task categories during 8 training sessions and 2 official games. A linear mixed model with random intercepts was used to compare training tasks and competition load, accounting for individual repeated measures. A 2-step cluster analysis was performed to classify the training tasks and games based on physiological and biomechanical load, employing log-likelihood as the distance measure and Schwartz’s Bayesian criterion.

**Results:**

Average heartrate , maximum heartrate, and high-speed skating (18.1-30 km/h) were the best physiological load predictors, while the most effective biomechanical load predictors were impacts [8-10] g(n), decelerations [-10 to -3]m/s²(n), and accelerations [3-10]m/s²(n). Different physiological and biomechanical responses were verified between training tasks and match demands. A 4-quadrant efforts assessment for each task category revealed that training tasks used by the team in the analysis presented lower biomechanical and physiological load demands than competition.

**Conclusion:**

Training tasks failed to adequately replicate the specific demands of competition, especially regarding high mechanical stress, such as the absence of high-intensity impacts and decelerations.

**Clinical Relevance:**

This method of classification of training tasks may allow coaches to understand further the specificity and contribution of each task to competition demands, consequently improving the capacity of load management and the preparedness and readiness of players for competition.

Team-sports, such as rink hockey, require players to perform several high-intensity efforts that include explosive technical movements, changes of direction, collisions, and short- and long-duration movements (eg, medium- and high-speed distance), followed by short recovery intervals, which impose significant physiological, biomechanical, and psychological demands.^12^ For players to respond effectively to these demands, coaches and sports scientists must adjust training sessions to improve, among other things, training specificity to competition demands,^2^ and the athlete readiness accordingly.^14,25^

In this regard, proper quantification of competition and training load (TL) is a critical part of the process as it allows (1) understanding of player performance during match-play; (2) determination of how well players adjust to training stimuli; and (3) assessment of whether training regimens actually resemble and replicate competition demands.^17,24^ Time-motion tracking and heartrate (HR) monitoring are commonly employed to quantify competition and TL through measures of external load (EL) and internal load (IL).^15^ The use of such measures allows the relationship between the dose and response to be understood and monitored to properly manage fatigue and recovery and promote player readiness for competition.^14,19^

According to Rossi,^28^ EL is considered as the dose and is usually categorized into 3 main groups based on its nature: (1) kinematics, which quantifies the total energy expended by movement during exercise (eg, total distance [m]); (2) mechanical, which describes the player’s overall load during exercise (eg, accelerations (n)); and (3) metabolic, which measures the total amount of energy expended during exercise (eg, metabolic power). Complementarily, IL is defined as the response and has been used to understand player physiological responses to EL,^3^ usually through rating of perceived exertion or HR measures.^1^

Particularly in rink hockey, recent research has been developed under this approach to assess the load of training and competition,^9-11^ mainly through the global characterization of EL and IL. For example, Fernández et al^10^ reported that high-speed skating (HSS >18 km/h), accelerations (ACC), and decelerations (DEC) were the EL variables that better characterized the most demanding efforts in this sport, and that match-day (MD)-4 and MD-1 sessions tended to place athletes in a “low EL and IL” zone. Conversely, in MD-3 and MD-2 sessions, as well as on MD, where greater loads were recorded (ie, ACC from 3 to 10 m/s^2^, DEC from -10 to -3 m/s^2^, and HSS >18 km/h), the majority of the players were found to be in a “high EL and IL” zone, with a tendency to disperse towards the fitness or tiredness zones.^10^ Notably, peak demands in rink hockey have been defined as position-dependent, with exterior players covering more distance and inside players performing more ACC, supporting the importance of using such metrics for the physical improvement of players with more exterior and interior tactical actions, respectively.^11^ Still, despite these studies, further research is needed to better understand the specificity of each training task considering the contribution of EL dimensions (ie, kinematic, mechanic, and metabolic) and their relationship with IL during competition.

Regarding training, previous rink hockey studies have described variations in load between and within microcycles.^11^ However, only a general description of the training session as a whole has been provided, without any reference to the specificity of the training tasks. Consequently, there is no clear understanding of the demands of each training task and its contribution to the weekly load, or even to replicate competitive demands. For instance, as already verified in other team-sports (ie, futsal),^27^ it is conceivable that different training exercises may have a different impact on player load and, consequently, on their preparation. As such, understanding the dose-response effects of exercises typically prescribed,^26^ and their specificity in relation to match demands,^11,21^ may contribute to the optimization of the load management and training process in general.

Therefore, this study aimed to propose an applied approach to characterizing and classifying training task specificity in relation to competition in a top-level rink hockey team.

Using a quadrant-based classification system, it was expected that this method would allow training tasks and games to be classified according to physiological and biomechanical load, assisting sports scientists and coaches in improving load management and defining the best exercises according to the required goal.

## Methods

### Study Design

An observational study was conducted during 2 consecutive microcycles (from March 21 to April 2, 2022) of a team competing in the First Division of the Portuguese Rink Hockey League. Since tracking system antennas are not permitted at an opponent’s pavilion, these 2 weeks were chosen based on the availability of the team and the competition schedule so as to monitor 2 sequential training microcycles and official home games. In total, 10 players participated in 8 training sessions and 2 official games during the 2 weeks of the study. A nonexperimental descriptive method was used to characterize the training drills of the different sessions (ie, MD-4; MD-3; MD-2; MD-1) and official games (ie, MD1; MD2). The exercises performed during the training sessions and games were split and categorized according to specific criteria^27^: introductory technique-activation exercises (INT); analytical situations (ANLT); midcourt exercises (20 × 20 m) (EX.MD); three-quarters of the court exercises (15 × 18 m) (EX.IN¾); full-court exercises (20 × 40 m) (EX.FC); superiorities/inferiorities (EX.S/I); and official games considering effective (GM.EFF) or running time (GM.RUN) (Table 1). During data collection, no instruction was given to technical staff regarding exercise selection or in-match coaching decisions.

**Table 1.** Task description for each category analyzed

Exercise Category		Task Description
INT	Introductory technique - activation exercises	✓ Skating activation with changes of direction ✓ Manipulate the stick with the ball, associating passes with/without opposition. ✓ 1 × 1 ✓ Upper- and lower-body activation ✓ Goalkeeper warm-up ✓ Strength exercises ✓ Proprioception exercises
ANLT	Analytical situations	✓ Analytical, tactical movements with/without opponents with passes, specific changes of direction, and finalization (ie, shooting) ✓ Changes of position while skating and passing, finishing with a shot on goal from different positions ✓ Shot on goal from the penalty spot/free kick
EX.MD	Midcourt exercises (20 × 20 m)	✓ Game with/without goalkeeper in 2 vs 2; 3 vs 3; and 4 vs 4 formats
EX.IN¾	Three-quarters of the court exercises (15 × 18 m)	✓ Game (1 vs 1and 2 vs 2) with/without goalkeeper
EX.FC	Full-court exercises (20 × 40 m)	✓ Game (3 vs 3 and 4 vs 4) ✓ Exercises involving both attacking and defensive actions (counterattack)
EX.S/I	Superiorities/inferiorities	✓ Situations with numerical superiorities/inferiorities
GM.EFF	Official game	✓ Effective time registered
GM.RUN	Official game	✓ Running time registered

### Participants

Data from a convenience sample of 10 elite-level male rink hockey players (4 defenders/midfielders and 6 forwards; age, 27.4 ± 5.5 years; height, 174.8 ± 2.8 cm; body mass, 74.92 ± 2.83 kg, body mass index, 24.53 ± 0.95 kg/m^2^) competing at the Portuguese elite national championship who participated voluntarily in the study were collected. Goalkeepers were not included in the analysis because of the different training categories and match demands. Players were considered for inclusion if they: (1) had no injury or court time limitation during the data collection period; (2) completed the entire training session or game. Throughout the data collection period, no athletes were dropped from the study.

### Ethics Statement

Data collection was carried out according to the international ethical standards with humans based on the Declaration of Helsinki.^16^ All participants signed written informed consent forms after being apprised of the protocol and the procedures. Participation was voluntary, and each participant could withdraw at any time. To ensure player confidentiality, all data were anonymized before analysis.

### Procedures

Data collection related to player activity during in-court sessions with skates was carried out with Inertial Movement Units (IMUs) using an ultra-wideband (UWB) local positioning system (LPS) technology from WIMU PRO (Realtrack Systems SL). HR data were collected by using a GARMIN HRM-PRO chest strap (Garmin Ltd) located below the chest. Also, training and games were recorded on video. All data from WIMU and GARMIN were downloaded using their corresponding software (SPRO, Realtrack Systems SL, Version 986) and synchronized with the video. The devices were turned on 5 to 10 minutes before the warm-up began and placed in a tight-fitting harness in the upper part of the athlete’s back. Six antennas with UWB technology were fixed ±5 m to the court. The LPS system works by triangulating the LPS unit devices and the antennas and determining the unit’s position (x and y coordinates) by using 1 of the antennas as a reference. The accuracy and reliability of the WIMU devices have been reported and validated previously.^3^ To evaluate the GM.EFF and GM.RUN, an interactive program (Breakaway Time Rink Hockey, Sports Performance Analytic Inc) was used to accurately calculate the time period during which players were actively engaged in the game versus the time when the game was paused.

From the data collected, EL and IL parameters were classified into physiological and biomechanical variables (Table 2).^6^ The physiological variables recorded were average HR, maximum HR, HSS, medium-speed skating, and total distance skated (TDS). The biomechanical variables consisted of: the number of high-intensity impacts; high-intensity DEC, high-intensity ACC, and player load (Table 2). To translate and contrast the demands of the overall load of official games and training sessions, data were expressed as relative values per minute (min^-1^).

**Table 2.** Physiological and biomechanical variables recorded in this study

Type	Variable	Unit	Description
Time of practice	Duration of the exercise/match	TMOP (m)	Total time of practice, minutes
Physiological	HR	HR_AVG_ (bpm)	Average HR, bpm
		HR_MAX_ (bpm)	Maximum HR, bpm
	Total distance skating	TDSmin^-1^ (m)	Total distance skated in meters
	Moderate-speed skating	MSSmin^-1^ (m)	Total distance skated between 12.1 and 18 km/h/min
	High-speed skating	HSSmin^-1^ (m)	Total distance skated between 18.1 and 30 km/h/min
Biomechanical	High-intensity impacts	HIMPCTSmin^-1^ (g)	Total number of impacts recorded between 8 and 10*g* per minute
	Player load	PLmin^-1^ (a.u.)	Accumulated accelerometer load in the 3 axes of movement per minute
	High-intensity ACC	ACCmin^-1^ (m)/ [3 10]m/s^2^ (n)	Total number of positive speed changes per minute
	High-intensity DEC	DECmin^-1^ (m) [-10 -3]m/s^2^ (n)	Total number of negative speed changes per minute

ACC, acceleration; DEC, deceleration; DECmin^-1^ (m), number of decelerations per minute; HIMPCTSmin^-1^ (g), high impacts per minute; HR, heartrate; HR_AVG_, average HR; HR_MAX_, maximum HR; HSSmin^-1^ (m), high-speed skating per minute; MSSmin^-1^ (m), medium-speed skating per minute; TDSmin^-1^ (m), total distance skated per minute; TMOP (min), time of practice.

### Statistical Analysis

Based on a previous power analysis through G*Power (3.1.9.2) (Cohen’s *d* effect size [ES] of 0.8, probability of error of 0.05, and power of 0.8), a sample size of 8 rink hockey players was required.^8^ Descriptive statistics (range, mean, standard error of the mean, and standard deviation) were calculated for the overall sample.

A linear mixed model with random intercepts was used to compare EL and IL differences between the training and match categories analyzed, accounting for individual repeated measures. External (TDSmin^-1^; MSSmin^-1^; HSSmin^-1^; HIMPCTSmin^-1^; ACCmin^-1^; DECmin^-1^) and internal (HR_AVG_ and HR_MAX_) loads were included as fixed effects, subjects were included as random effects, and the training and game categories were treated as a continuous variable. The distribution of the residuals was examined after fitting a linear mixed model.^4^ Cohen’s *d* ES and 95% CI were calculated and interpreted as follows: <0.2 trivial; 0.20 to 0.59 small; 0.60 to 1.19 moderate; 1.2 to 1.99 large; and ≥2.0 very large.^18^ The significance level was set at *P* < 0.05. In addition, a 2-step cluster analysis was performed to classify the training and game categories based on physiological and biomechanical load, employing log-likelihood as the distance measure and Schwartz’s Bayesian criterion.^22^ Finally, to determine the physiological load of each training task, an average was calculated for each training and game category based on the numerical load value of the cluster ranging from 1 to 2. The same was performed to determine the biomechanical load based on the biomechanical numerical load value of the cluster ranging from 1 to 3.

The statistical analysis was conducted using the statistical package Jamovi (Version 1.8, 2021) for the linear mixed model analysis and IBM SPSS Statistics for Windows (Version 28.0, IBM Corp) for the 2-step cluster analysis.

## Results

Appendix 1, available in the online version of this article, presents the descriptive statistics and the linear mixed model analysis results for the physiological and biomechanical variables measured in each training and game category. For all physiological and biomechanical variables, GM.EFF and GM.RUN showed significantly (*P* < 0.01) higher values than all the training tasks. Despite significant differences observed among almost all categories of training, EX.MD and EX.FC exhibited consistently higher values (*P* < 0.01) across all physiological and biomechanical variables.

Regarding the physiological impact, INT and EX.IN¾ training tasks displayed low physiological impact in HR_AVG_ and HR_MAX_ in comparison with other tasks. Also, EX.IN¾ and EX.S/I displayed the lowest values of TDS and MSSmin^-1^, and INT and EX.S/I training tasks showed lower HSSmin^-1^. Finally, GM.EFF and GM.RUN showed significantly higher values for all physiological metrics except for HR_AVG_ between EX.MD and GM.RUN (see Appendix 1, available online).

When considering the biomechanical impact, several variations occurred. For the training tasks INT, ANLT, EX.IN¾, EX.S/I, lower values of DECmin^-1^, ACCmin^-1^, and PLmin^-1^ were observed in comparison with the EX.MD and EX.FC tasks (Appendix 1, online). GM.EFF and GM.RUN showed significantly (*P* < 0.01) higher values of HIMPCTmin^-1^, DECmin^-1^, ACCmin^-1^, and PLmin^-1^ than all the training tasks. EX.MD and EX.FC consistently displayed higher values of PLmin^-1^ (a.u.) (*P* < 0.01) in relation to other training tasks except for EX.S/I. Finally, GM.EFF tended to exhibit moderately higher (*P* ≤ 0.05) biomechanical impact (ie, DECmin^-1^, ACCmin^-1^, and PLmin^-1^) than GM.RUN. No differences were observed between training tasks for HIMPCTSmin^-1^.

The cluster analysis classified the physiological parameters into 2 distinct groups: “low physiological load” and “high physiological load.” Both clusters were strongly related to the predictor variables, with HR_AVG_, HR_MAX_, HSSmin^-1^, MSSmin^-1^, and TDSmin^-1^ having predictor’s importance of 1.00, 0.76, 0.74, 0.57, and 0.47, respectively. The average silhouette measure of cohesion and separation (representative of the clustering quality) was 0.5, which indicated a moderate model quality (Table 3).

**Table 3.** Cluster analysis identifying exercise groups based on physiological and biomechanical load variables

Cluster	Variable	Importance of the Cluster Predictor	Low Physiological Load		High Physiological Load
Physiological Cluster	HR_AVG_, bpm	1.00	117.96 ± 17.09		149.25 ± 14.28
	HR_MAX_, bpm	0.76	138.68 ± 19.34		166.61 ± 13.90
	HSSmin^-1^, m	0.74	2.91 ± 3.80		11.19 ± 6.67
	MSSmin^-1^, m	0.57	15.94 ± 11.93		39.46 ± 23.37
	TDSmin^-1^, m	0.47	79.94 ± 36.23		132.56 ± 36.23
	Sample size, N		533		333
	Sample percentage		61.5%		38.5%
	BIC		2287.52		
	Average silhouette		0.5		
Cluster	Variable	Importance of the Cluster Predictor	Low Biomechanical Load	Medium Biomechanical Load	High Biomechanical Load
Biomechanical Cluster	HIMPCTSmin^-1^, g	1.00	0.01 ± 0.02	0.03 ± 0.06	0.27 ± 0.15
	DECmin^-1^, m	0.96	0.98 ± 0.67	3.05 ± 1.25	4.81 ± 2.54
	ACCmin^-1^, m	0.81	1.18 ± 0.74	3.28 ± 1.48	5.98 ± 3.81
	PLmin^-1^, a.u.	0.67	0.42 ± 0.13	0.63 ± 0.17	0.95 ± 0.39
	Sample size, N		455	359	52
	Sample percentage		52.5%	41.5%	6.0%
	BIC		1428.15		
	Average silhouette		0.5		

BIC, Bayesian information criterion; DECmin^-1^ (m), number of decelerations per minute; HIMPCTSmin^-1^ (g), high impacts per minute; HR, heartrate; HR_AVG_, average HR; HR_MAX_, maximum HR; HSSmin^-1^ (m), high-speed skating per minute; MSSmin^-1^ (m), medium-speed skating per minute; TDSmin^-1^ (m), total distance skated per minute.

Regarding biomechanical load, cluster analysis classified the categories into 3 distinct groups: (1) “low biomechanical load,” (2) “medium biomechanical load,” and (3) “high biomechanical load.” The clusters displayed a strong association with the predictor variables, HIMPCTSmin^-1^, DECmin^-1^, ACCmin^-1^, and PLmin^-1^, having a predictor’s importance of 1.00, 0.96, 0.81, and 0.67, respectively. The average silhouette was 0.5, indicating a moderate model quality (Table 3).

Based on the previously performed cluster analysis by training and game categories, Table 4 provides the descriptive physiological and biomechanical average results. GM.EFF and GM.RUN showed higher physiological and biomechanical clusters average, followed by EX.FC and EX.MD. Finally, INT, ANLT, EX.IN¾, and EX.S/I were the exercises with lower physiological and biomechanical values.

**Table 4.** Descriptive analysis of physiological and biomechanical load for each training and match category

Training and Match Categories	Physiological Load		Biomechanical Load	
	Mean ± SD	Median	Mean ± SD	Median
Introductory technique - activation exercises	1.13 ± 0.34	1	1.23 ± 0.42	1
Analytical situations	1.31 ± 0.46	1	1.45 ± 0.53	1
Exercises in midcourt (20 × 20 m)	1.70 ± 0.46	2	1.72 ± 0.50	2
Exercises in ¾ of the court (15 × 18)	1.14 ± 0.35	1	1.48 ± 0.59	1
Exercises in full court (20 × 40 m)	1.78 ± 0.41	2	1.76 ± 0.51	2
Superiorities/inferiorities	1.21 ± 0.41	1	1.31 ± 0.53	1
Official game (effective time)	2.00 ± 0.00	2	2.94 ± 0.23	3
Official game (running time)	2.00 ± 0.00	2	2.72 ± 0.46	3

**Figure 1.**
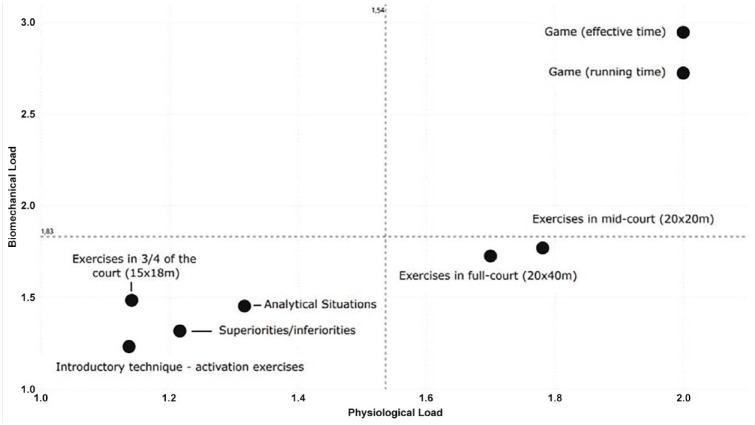
Distribution quadrants of the physiological and biomechanical requirements of training exercises and the game demands.

Considering the results from Table 4, each training and game category was classified according to the calculation of the mean value of the classification of each player in each training task. The quadrant classification (Figure 1) showed that GM.EFF and GM.RUN were the unique categories that presented high biomechanical and physiological efforts. EX.FC and EX.MD were characterized as medium-high physiological efforts and low-medium biomechanical efforts. Finally, INT, ANLT, EX. IN¾, and EX. S/I were characterized as low physiological and biomechanical efforts. Interestingly, no exercise was described as a high biomechanical and low physiological effort.

## Discussion

The aim of this study was to propose an applied approach to characterize and classify the training task specificity in relation to competition in a top-level rink hockey team. Training tasks did not meet the specificity of the competition load. The physiological and biomechanical metrics for GM.EFF and GM.RUN indicated higher demands compared with all training tasks. Furthermore, in the training tasks, EX.MD and EX.FC exhibited significantly greater physiological and biomechanical metrics compared with the other exercises. Considering its structure and specificity, these findings provide valuable insights into the load management of rink hockey exercises and their integration in the microcycle.

Despite the concept that training tasks and sessions should be equivalent, or even superior, to game demands, previous research has shown that rink hockey training does not replicate the physical exigencies players experience during game-play.^11^ Indeed, even though MD-3 and MD-2 have already been described as the most demanding training sessions (with the highest EL and IL of the microcycle), the training demands are still lower than those of a game, compromising the specificity of the training and the preparedness of the players to face the demands of competition.^10,11,13^ Despite evidence regarding the differences between overall training and game demands, to the best of our knowledge, this is the first study to describe, in depth, the specificity of training tasks in relation to competitive demands. This novel evidence-based analysis approach for quantifying the load in elite rink hockey is detailed through relative values (per minute), outlining the compilation of EL and IL responses across various task types compared with the demands observed during games. Therefore, the results herein may provide important information and contribute to a more comprehensive understanding of the impact of each training task on the preparedness of rink hockey players to face competition.^13^

It is well established that team-sport training and competition promote physiological adaptations that improve athletic performance by contributing to the development of endurance, speed, and power.^28^ Thus, identifying which metrics may lead to greater adaptations is relevant for the training process. The method presented in this study allowed us to classify each drill according to the level of physiological (ie, low- and high-physiological load) and biomechanical (ie, low-, medium-, and high-biomechanical load) exigency and, at the same time, to clarify which variables had a greater impact on such clustering. Results show that HR_AVG_, HR_MAX_, and HSS have a strong influence on rink hockey training and game physiological loads, whereas HIMPCTS, DEC, and ACC seem to be the main determinants of the biomechanical demands of this sport. This is the first time these metrics have been presented in terms of a cluster predictor’s relevance, which may be helpful for coaches. Based on the present data, practitioners may adjust their programming (ie, exercise selection) depending on the need to increase or decrease the physiological and biomechanical stress imposed on their players in each training session, as has been already proposed in different team-sports, such as futsal and basketball.^23,26^ For example, in practical settings, if rink hockey coaches want to impose a high physiological stress on their athletes, EX.MD (eg, 2 vs 2, 3 vs 3, or 4 vs 4 formats in a 20 × 20 m area) may be the most suitable option.

Notably, one of the most relevant findings of the present study was that no training task was placed on the “high-biomechanical load/high physiological load” quadrant as occurs with the GM.EFF and GM.RUN. Moreover, no training task was characterized by simultaneously having low physiological and high biomechanical loads (Table 4 and Figure 1). In line with the low predictor’s importance on the cluster analysis, the low incidence of HIMPCTS and DEC during training may have promoted a reduction in the biomechanical load experienced by the players. Based on previous research, mechanical training stress has the ability to promote adaptations related to “fitness” or “fatigue.”^6^ Therefore, it appears that modifying training task characteristics to increase the frequency of efforts that imposed high mechanical stress during training (eg, HIMPCTS and DEC) might be a good strategy to better prepare rink hockey players to endure game-play demands.^25^ In addition, the lack of specific tasks with high HIMPCTS and DEC counts may lead to lower levels of unpredictability and fatigue-inducing activities in open sports like rink hockey.^6^ In this regard, differences in the specificity between the training environment and game-play are important to account for since they may be associated with a higher level of discomfort, mental exhaustion, and greater risk of injuries during competition.^6,13^ As argued by some authors, the main reason why injuries in team sports are higher in competitive settings is due to the unmatched physiological demands in training sessions.^5,7^ Interestingly, our results may bring a new perspective to this matter by showing that, in elite rink hockey, training drills do not replicate competition demands in terms of their biomechanical load. This is a crucial aspect to consider because, as in other contact sports, mechanical metrics such HIMPCTS and DEC play an important role in the characterization of game-play.^15,20^ Therefore, according to the present data, it could be argued that proper monitoring and manipulation of the biomechanical load in rink hockey training drills is fundamental to protect players from possible postcompetition muscle damage as a consequence of HIMPCTS and DEC (ie, eccentrically biased efforts by nature). This approach may also conceivably enhance athlete performance by enabling coaches to track relevant data during training cycles, thus, effectively managing the dynamics of the load to ensure better preparedness for the physical requirements of a game. Also, results and methodology herein may be used as benchmark for training monitoring in rink hockey to promote an innovative vision of microcycle manipulation and load management. Furthermore, the classification of each training task according to a quadrant’s representation is a novel approach that may help practitioners better comprehend, visualize, and evaluate the appropriateness of typical rink hockey drills by having game-play demands as a reference for the physiological and biomechanical demands to which players may be submitted.

### Practical Applications

The present findings may assist trainers and strength and conditioning coaches to bridge the gap between training and competition, improving the specificity of training tasks and routines to improve players’ preparedness for competition. Moreover, the proposed categorization of exercises into quadrants (Figure 1) can be a valuable tool for optimizing training plans and load management in elite rink hockey. From a practical perspective, the current results indicate that, if the aim is to impose physiological loads similar to those observed in competition, coaches should use training tasks such as EX.MD (eg, game with/without goalkeeper in 2 vs 2; 3 vs 3; and 4 vs 4 formats) or EX.FC (eg, exercises involving both attacking and defensive actions and counterattacks) that elicit great HR_AVG_ and HR_MAX_ responses and that include high HSS distances. Conversely, if the goal is to replicate the demands of competition in terms of biomechanical loading, coaches should use training tasks such as conditioned games (number of players, time of play, and tactical constraints) in midcourt, that elicit a higher number of HIMPCTS, DEC, and ACC efforts.

### Limitations

Major limitations must be addressed in this study. The fact that only 1 team of 10 players and 1 coaching staff were involved in this study during 2 microcycles severely limits the generalization of the findings with respect to the demands of the specific drills presented. Also, the moment of the season or the type of competition (eg, European competition vs domestic competition) were not considered variables.

## Conclusion

Game-play physiological and biomechanical loads are significantly greater than all training drills. The quadrant assessment for each exercise category revealed that only GM.EFF and GM.RUN were characterized by having high biomechanical and physiological loads. The physiological efforts of EX.FC and EX.MD were medium to high, whereas the biomechanical efforts were low to medium. Finally, exercises such as INT, ANLT, EX.IN¾, and EX.S/I were found to place minimal physiological and biomechanical demands on players when compared with game-play. The current methodology constitutes an innovative way to monitor rink hockey loads by classifying and adjusting specific exercises, using game-play demands as a reference.

## Supplemental Material

sj-pdf-1-sph-10.1177_19417381241273219 – Supplemental material for Bridging the Gap Between Training and Competition in Elite Rink Hockey: A Pilot Study Supplemental material, sj-pdf-1-sph-10.1177_19417381241273219 for Bridging the Gap Between Training and Competition in Elite Rink Hockey: A Pilot Study by António Ferraz, Enrique Alonso Pérez-Chao, João Ribeiro, Konstantinos Spyrou, Tomás T. Freitas, João Valente-dos-Santos, Pedro Duarte-Mendes, Pedro E. Alcaraz and Bruno Travassos in Sports Health
