# Supplementary material for: Bridging the Gap Between Training and Competition in Elite Rink Hockey: A Pilot Study
Source: Sports Health. 2024 Aug 27;17(1):57–65. doi: 10.1177/19417381241273219 (PMC11569628; doi:10.1177/19417381241273219)
Supplement: sj-pdf-1-sph-10.1177_19417381241273219 – Supplemental material for Bridging the Gap Between Training and Competition in Elite Rink Hockey: A Pilot Study [file sj-pdf-1-sph-10.1177_19417381241273219.pdf]

Appendix 1. Linear Mixed Model and Description of the Physiological and Biomechanical metrics by training and match categories

Variables	Exercise Categories												Game				
	**INT**<br>***n*=160**<br>**(95% CI)**	**ES**	**ANLT**<br>***n*=290**<br>**(95% CI)**	**ES**	**EX.MD**<br>***n*=40**<br>**(95% CI)**	**ES**	**EX. IN¾**<br>***n*=120**<br>**(95% CI)**	**ES**	**EX. FC**<br>***n*=160**<br>**(95% CI)**	**ES**	**EX. S/I**<br>***n*=60**<br>**(95% CI)**	**ES**	**GM.EFF**<br>***n*=18**<br>**(95% CI)**	**ES**	**GM.RUN**<br>***n*=18**<br>**(95% CI)**	**ES**	**Total**<br>***n*=866**
TMOP (min)	6.68±4.25<br>[6.01;7.33]		5.21±3.12<br>[4.85;5.57]		6.15±2.59<br>[5.32;6.98]		5.20±2.84<br>[4.69;5,71]		10±4.65<br>[9.32;10.77]		4.80±1.24<br>[4.48;5.12]		23.1±10.70<br>[17.77;28.46]		31.7±15.00<br>[25.23; 39.73]		7.23±6.39<br>[6.80;7.66]
Physiological																	
HR_{AVG} (bpm)	**** # Δ α β Φ ¥**<br>113.67±17.31<br>[110.96;116.37]	0.6<br>0.8<br>0.4<br>1.2<br>0.4<br>0.8<br>0.8	**# α Φ ¥**<br>126.59±16.95<br>[124.63;128.55]	0.5<br>0.8<br>0.6<br>0.6	**Δ βΦ**<br>147.65±16.79<br>[142.28;153,02]	0.5<br>0.4<br>0.2	**α Φ ¥**<br>124.72±16.36<br>[121.76;127.67]	0.7<br>0.6<br>0.6	**β Φ ¥**<br>144.92±25.07<br>[141.00;148.83]	0.4<br>0.3<br>0.3	**Φ ¥**<br>129.95±14.64<br>[126.17;133.73]	0.5<br>0.5	163.06±10.72<br>[157.72;168.39]		160.06±11.24<br>[154.47;165.64]		129.99±22.13<br>[128.52;131.46]
HR_{MAX} (bpm)	**** # Δ α β Φ ¥**<br>133.24±21.69<br>[129.85;136.63]	0.5<br>0.7<br>0.4<br>1.2<br>0.5<br>0.8<br>0.8	**# α Φ ¥**<br>144.64±16.70<br>[142.72;146.57]	0.5<br>0.9<br>0.6<br>0.6	**Δ β Φ ¥**<br>164.35±15.01<br>[159.55;169.15]	0.4<br>0.3<br>0.3<br>0.3	**α Φ ¥**<br>144.82±16.06<br>[141.91;147.72]	0.7<br>0.6<br>0.6	**β Φ ¥**<br>166.03±21.89<br>[162.61;169.45]	0.4<br>0.3<br>0.3	**Φ ¥**<br>151.53±12.32<br>[148.35;154.72]	0.5<br>0.5	181.17±8.51<br>[176.93;185.40]		181.17±8.51<br>[176.93;185.40]		149.42±22.12<br>[147.91;150.89]
$HSSmin^{-1}(m)$	**** # α Φ ¥**<br>1.29±2.54<br>[0.89;1.68]	0.3<br>0.4<br>1.3<br>2.6<br>2.6	**α Φ ¥**<br>5.90±6.76<br>[5.12;6.68]	1.1<br>2.5<br>2.5	**α Φ ¥**<br>7.35±4.75<br>[5.83;8.87]	0.5<br>2.0<br>2.0	**α Φ ¥**<br>5.23±5.02<br>[4.31;6.14]	1.0<br>2.4<br>2.4	**β Φ ¥**<br>9.98±6.40<br>[8.98;10.97]	0.8<br>2.0<br>2.0	**Φ ¥**<br>4.46±4.43<br>[3.31;5.61]	2.3<br>2.3	18.14±4.33<br>[15.99;20.30]		13.85±3.79<br>[11.97;15.74]		6.09±6.49<br>[5.67;6.52]
$MSSmin^{-1}(m)$	**# α Φ ¥**<br>19.87±31.22<br>[15.00;24.75]	0.3<br>0.4<br>0.7<br>0.4	**α Φ ¥**<br>23.53±14.80<br>[21.82;25.24]	0.3<br>0.6<br>0.4	**Δ β Φ¥**<br>32.09±12.27<br>[28.17;36.02]	0.3<br>0.3<br>0.4<br>0.2	**α Φ ¥**<br>17.81±8.08<br>[16.35;19.27]	0.4<br>0.7<br>0.5	**β Φ ¥**<br>31.56±18.46<br>[28.68;34.45]	0.3<br>0.5<br>0.3	**Φ ¥**<br>18.12±15.72<br>[14.06;22.19]	0.6<br>0.4	65.66±12.98<br>[59.21;72.12]		49.38±14.36<br>[42.24;56.52]		24.98±20.69<br>[23.60;26.36]
$TDSmin^{-1}$ (m)	**Δ Φ ¥**<br>99.89±56.92<br>[91.00;108.78]	0.2<br>0.9<br>0.5	**α Φ ¥**<br>89.07±32.84<br>[85.28;92.87]	0.4<br>1.0<br>0.5	**Δ β Φ ¥**<br>118.67±21.45<br>[111.81;125.53]	0.3<br>0.3<br>0.7<br>0.3	**α Φ ¥**<br>82.24±27.01<br>[77.36;87.13]	0.4<br>1.0<br>0.6	**β Φ ¥**<br>112.16±40.30<br>[105.87;118.45]	0.3<br>0.8<br>0.4	**Φ ¥**<br>82.78±25.63<br>[76.16;89.40]	0.9<br>0.5	**¥**<br>238.71±125.35<br>[176.38;301.06	0.3	172.69±87.07<br>[129.39; 215.99]		100.17±50.36<br>[96.82;103.52]
Biomechanical																	
$HIMPCTSmin^{-1}(g)$	Φ¥<br>0.01±0.03<br>[0.001;0.010]	1.0<br>0.7	**Φ¥**<br>0.02±0.07<br>[0.01;0.03]	0.9<br>0.6	**Φ ¥**<br>0.03±0.06<br>[0.01;0.05]	0.8<br>0.5	**Φ¥**<br>0.02±0.09<br>[0.008;0.040]	0.9<br>0.6	**Φ¥**<br>0.04±0.07<br>[0.03;0.05]	0.9<br>0.6	**Φ¥**<br>0.02±0.06<br>[0.001;0.035]	0.9<br>0.6	0.26±0.17<br>[0.17;0.34]		0.18±0.13<br>[0.12;0.25]		0.03±0.08<br>[0.02; 0.04]
$DECmin^{-1}(m)$	**** # Δ α Φ ¥**<br>1.26±1.65<br>[0.99;1.52]	0.3<br>0.4<br>0.4<br>0.5<br>1.0<br>1.1	**α Φ ¥**<br>1.85±1.43<br>[1.68;2.02]	0.3<br>0.9<br>0.6	**β Φ ¥**<br>2.56±1.13<br>[2.19;2.92]	0.2<br>0.7<br>0.4	**Φ ¥**<br>2.18±1.60<br>[1.89;2.47]	0.8<br>0.5	**β Φ ¥**<br>2.48±0.87<br>[2.34;2.62]	0.3<br>0.8<br>0.5	**Φ ¥**<br>1.53±0.85<br>[1.31;1.75]	0.9<br>0.6	**¥**<br>6.44±2.67<br>[5.11;7.77]	0.2	5.51±2.81<br>[3.74;5.61]		2.06±1.65<br>[1.95;2.17]
$ACCmin^{-1}$ (m)	**** # Δ α Φ ¥**<br>1.07±1.32<br>[0.87;1.28]	0.6<br>0.5<br>0.5<br>0.6<br>1.2	**Φ ¥**<br>1.42±1.77<br>[2.19;2.60]	1.0<br>0.7	**β Φ ¥**<br>2.90±1.55<br>[2.41;3.40]	0.3<br>0.8<br>0.5	**β Φ ¥**<br>2.41±1.47<br>[2.14;2.67]	0.2<br>1.0<br>0.7	**β Φ ¥**<br>2.46±0.90<br>[2.33;2.60]	0.3<br>1.0<br>0.7	**Φ ¥**<br>1.59±0.92<br>[1.35;1.83]	1.1<br>0.8	**¥**<br>8.12±4.06<br>[6.10;10.13]	0.2	7.40±4.28<br>[4.71;8.07]		2.33±1.98<br>[2.20;2.46]
$PLmin^{-1}$(a.u.)	**# α Φ ¥**<br>0.46±0.19<br>[0.43;0.49]	0.9<br>0.4<br>0.4<br>1.2<br>0.8	**# α £ Φ ¥**<br>0.49±0.16<br>[0.47;0.51]	0.3<br>0.4<br>1.2<br>0.7	**Δ Φ ¥**<br>0.64±0.16<br>[0.59;0.69]	0.3<br>0.8<br>0.4	**# α Φ ¥**<br>0.49±0.14<br>[0.46;0.52]	0.3<br>1.1<br>0.7	**Φ ¥**<br>0.58±0.23<br>[0.55;0.62]	1.0<br>0.6	**Φ ¥**<br>0.53±0.15<br>[0.49;0.57]	1.0<br>0.6	**¥**<br>1.24±0.37<br>[1.05;1.42]	0.3	0.96±0.30<br>[0.81;1.11]		0.54±0.23<br>[0.52;0.55]

Data presented as mean ± standard deviation and *IC*

Time of Practice (TMOP (min)); Heart rate average (HR_{AVG}; Maximum heart rate (HR_{MAX}); High-speed skating per minute ($HSSmin^{-1}(m)$); Medium-speed skating per minute ($MSSmin^{-1}(m)$); Total distance skated per minute ($TDSmin^{-1}$ (m)); High Impacts per minute ($HIMPCTSmin^{-1}(g)$); Number of Decelerations per minute ($DECmin^{-1}(m)$).

** significantly different than ANLT ($p \leq 0.05$); # significantly different than EX.MD ($p \leq 0.05$); Δ significantly different than EX. IN¾ ($p \leq 0.05$); α significantly different than EX. FC ($p \leq 0.05$); β significantly different than EX.S/I ($p \leq 0.05$); Φ significantly different than GM.EFF ($p \leq 0.05$); ¥ significantly different than GM.RUN ($p \leq 0.05$); ES: Cohen's d effect size, reported only for significant differences and not reported for repeated significant differences. Repeated statistical differences between groups were not presented.
